# Molecular detection of *Coxiella burnetii* in livestock farmers and cattle from Magdalena Medio in Antioquia, Colombia

**DOI:** 10.1371/journal.pone.0234360

**Published:** 2020-06-10

**Authors:** Ruth Cabrera Orrego, Leonardo Alberto Ríos-Osorio, Yoav Keynan, Zulma Vanessa Rueda, Lina Andrea Gutiérrez

**Affiliations:** 1 Escuela de Ciencias de la Salud, Facultad de Medicina, Grupo Biología de Sistemas, Universidad Pontificia Bolivariana, Medellín, Antioquia, Colombia; 2 Escuela de Microbiología, Grupo de Investigación en Microbiología Veterinaria, Universidad de Antioquia, Medellín, Colombia; 3 Department of Internal Medicine, Medical Microbiology & Infectious Diseases and Community Health Sciences, University of Manitoba, Winnipeg, Canada; 4 Escuela de Ciencias de la Salud, Facultad de Medicina, Grupo de Investigación en Salud Pública, Universidad Pontificia Bolivariana, Medellín, Antioquia, Colombia; University of Lincoln, UNITED KINGDOM

## Abstract

*Coxiella burnetii* causes Q fever in humans and coxiellosis in animals. In humans, it causes acute febrile illnesses like influenza, pneumonia, hepatitis, and chronic illnesses such as endocarditis, vascular infection, and post-infectious fatigue syndrome. It is widely distributed worldwide, and its main reservoirs are sheep, goats, and cattle. This study aimed to determine the frequency of *C*. *burnetii* infection using molecular detection and to identify the associated factors in livestock farmers and cattle from the Magdalena Medio region of Antioquia, Colombia. Using real-time polymerase chain reaction (PCR), molecular detection was performed for the IS1111 insertion sequence of *C*. *burnetii* using genomic DNA collected from the peripheral blood of 143 livestock farmers and 192 cattle from 24 farms located in Puerto Berrío, Puerto Nare, and Puerto Triunfo. To confirm the results, bidirectional amplicon sequencing of *16S rRNA* was performed in four of the positive samples. Additionally, factors associated with *C*. *burnetii* were identified using a Poisson regression with cluster effect adjustment. Real-time PCR showed positive results in 25.9% and 19.5% of livestock farmer samples and cattle samples, respectively. For livestock farmers, factors associated with *C*. *burnetii* were the area where the farm was located [Puerto Berrío, adjusted prevalence ratio (aPR): 2.13, 95% confidence interval (CI): 1.10–4.11], presence of hens (aPR: 1.47, 95% CI: 1.21–1.79), horses (aPR: 1.61, 95% CI: 1.54–1.67), and ticks (aPR: 2.36, 95% CI: 1.03–5.42) in the residence, and consumption of raw milk (aPR: 1.47, 95% CI: 1.26–1.72). For cattle, the factors associated with *Coxiella* genus were municipality (Puerto Nare; aPR: 0.39, 95% CI: 0.37–0.41) and time of residence on the farm (≥49 months; aPR: 2.28, 95% CI: 1.03–5.20). By analyzing sequences of the *16S rRNA* molecular marker, *C*. *burnetii* infection was confirmed in livestock farmers. However, in cattle, only the presence of *Coxiella*-type bacteria was identified. Further research is necessary to determine the potential role that these types of bacteria have as etiological agents for disease in livestock farmers and cattle from the study area.

## Introduction

*Coxiella burnetii* is a zoonotic bacterium distributed worldwide (except in New Zealand) that causes a disease known as Q fever in humans and coxiellosis in animals [1,2]. Domestic ruminants (i.e., sheep, goats and cattle) are considered to be the main source of infection and are usually associated with outbreaks of infection in humans [3,4]. Infected animals, even if asymptomatic, can spread this bacterium to the environment through body fluids such as milk, urine, stool, cervical mucus, and fluids discharged at the time of parturition. Consequently, animals can pass the infection to their offspring, thereby promoting the transmission of this bacterium in productive cycles [5].

*C*. *burnetii* has been associated with Q fever outbreaks ever since it was first described as the causative agent of fever in slaughterhouse employees in Australia [2]. The disease is generally transmitted through contact with infected animals or their biological fluids; additionally, factors such as a large number of infected animals, farms located in close proximity to populated areas, and lack of epidemiological surveillance increase the incidence of infection [6]. Between 2007 and 2010, the largest Q fever outbreak reported to date involved more than 4,000 cases among animals and humans in the Netherlands [7]. Subsequently, it was demonstrated that appropriate surveillance, along with the integration of human and veterinary healthcare systems, could eradicate this disease [8]. Therefore, it is important to actively search for *C*. *burnetii* in livestock as a preventive measure.

Livestock production, especially cattle production, is crucial to the global economy [9]. When considering the characteristics of *C*. *burnetii* transmission, its association with cattle livestock has been recognized [10]. Within livestock production, the identified risk factors for human infection are those that require direct or indirect contact with animals and/or animal products and such activities include milking, servicing of enclosure, feeding, vaccination, assistance during birthing, dehorning, and treatment of infected animals [10,11].

In humans, up to 40% of infected individuals develop acute Q fever, which can evolve in three ways: a self-limiting febrile illness (similar to a common cold), pneumonia, or hepatitis [12–14]. Furthermore, approximately 2% of individuals who were exposed to the bacteria are estimated to develop chronic Q fever, which includes endocarditis, vascular infection, and post-infectious fatigue syndrome [15,16].

In Colombia, the presence of this bacterium was first described in 2006 in the departments of Córdoba and Sucre [17]; 23.6% (17/72) of the individuals assessed presented specific antibodies against *C*. *burnetii* (17). Subsequently, clinical cases of acute Q fever have been described [18], together with cases of endocarditis [19] and pneumonia [20]. In 2018, the results of a serological screening performed on anti *C*. *burnetii* IgG antibodies acquired from individuals exposed to livestock and cattle from the Northern and Magdalena Medio regions of Antioquia were published [21], which suggested that exposure occurs in that area of the country and performing a more exhaustive epidemiological surveillance that includes molecular analysis, in humans and animals, to confirm exposure to this microorganism is important. In this regard, the present study was conducted to determine the frequency of *C*. *burnetii* infection using molecular detection and assessing factors associated with its presence in livestock farmers and cattle from the Magdalena Medio region of Antioquia, Colombia.

## Methodology

### Ethics statement

This study was approved by the Ethics Committee on Health Research of the Universidad Pontificia Bolivariana (Record No. 7, May 23, 2012). Moreover, all individuals included in the present study provided informed consent, and consent for cattle sampling was obtained from the owner or land administrator.

### Area of study and population

**Fig 1:**
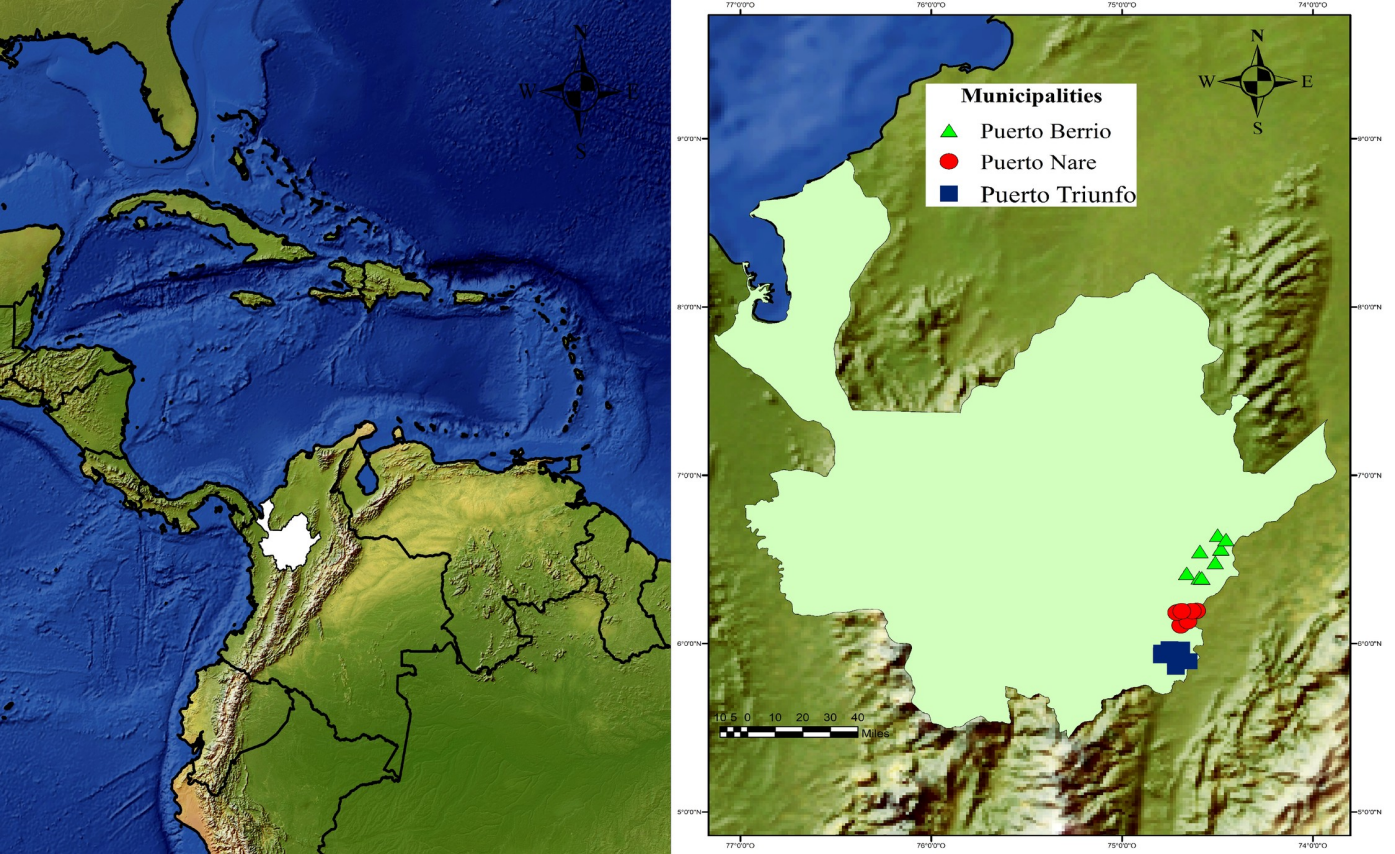
Geographic location of the livestock farms from the Magdalena Medio region of Antioquia.

This cross-sectional study was performed in 24 farms, eight farms per municipality, in the Magdalena Medio region of Antioquia, Colombia between October and November 2015 (Fig 1). A multistage sampling procedure was used. During the first stage, convenience samples from three local municipalities (i.e., Puerto Berrío, Puerto Nare, and Puerto Triunfo) were selected with temperatures ranging from 32°C to 43°C, 53% relative humidity, and altitudes from 125 to 150 MASL. These environmental conditions were verified during field sampling using a portable weather station (Ambient WS-2080; Ambient Weather, Chandler, USA). Subsequently, eight livestock herds were selected from each municipality (Fig 1) with a productive system of 176 cattle or higher to ensure availability of at least eight employees per farm (of both sexes and of legal age) who were working at the time of sampling. About the cattle, eight animals (regardless of its sex or age) were also included per farm.

A total of 143 individuals and 192 animals from the Magdalena Medio region of Antioquia were evaluated. Blood samples from cattle and livestock farmers were collected from the 24 farms, which are shown as green triangles for Puerto Berrío, red dots for Puerto Nare and blue squares for Puerto Triunfo. Geographic coordinates were determined during fieldwork using GPS Trimble Juno 3B equipment (Westminster, USA), and the map was represented using ArcGIS® 10.2 software Copyright© 1999–2013 Esri Inc. (http://www.esri.com/software/arcgis/index.html) with layers that are publicly available from Natural Earth (www.naturalearthdata.com).

To obtain blood samples, we extracted blood from the coccygeal vein of the tail-head of cattle and from a superficial vein, generally the median cubital vein, in the arm of farmers. Blood was extracted with a double-pointed needle into an anticoagulant vacutainer tube.

### Molecular diagnosis of *C*. *burnetii* in blood samples of livestock farmers and cattle

#### Molecular detection of C. burnetii using real-time PCR

Blood samples were collected in EDTA-containing tubes and stored at 4°C until delivered to the laboratory. Genomic DNA was then extracted using the salting-out protocol for small blood volumes [22]. The DNA concentration was assessed using a Nanodrop 2000 (Thermo Scientific, Massachusetts, USA), and as an internal and quality control of DNA (detection of PCR inhibitors), all samples were subjected to real-time PCR to detect the housekeeping gene encoding the GAPDH enzyme 5′-TGGGTGTGAACCATGAGAAG-3′ was used as the forward primer, and 5′-GCTAAGCAGTTGGTGGTGC-3′ was used as the reverse primer [23] with 5x HOT FIREPol® EvaGreen® qPCR Mix Plus (Solis BioDyne, Estonia), that comprises HOT FIREPol® DNA Polymerase, ultrapure dNTPs, MgCl2, EvaGreen® dye and ROX dye. Samples were positive when they showed an exponential amplification curve (Ct) up to the 30th cycle and peak of the melt curve between 80°C and 81°C.

For *C*. *burnetii* identification, the IS1111 insertion sequence was amplified via real-time PCR using 5′-AATTTCATCGTTCCCGGCAG-3′ as the forward primer, 5′-GCGGCGTTTACTAATCCCCA-3′ as reverse primer, and 5′-FAM-TGTCGGCGTTTATTGG-MGB-3′ as a probe, all of which were synthesized by Integrated DNA Technologies, Inc. (Coralville, Iowa, USA). Amplifications were performed using TaqManFast Universal PCR Master Mix (2x) kit (that contains AmpliTaq Fast DNA Polymerase and all of the components to perform a real time PCR, excluding the water, template, primers and probe) in an Applied Biosystems 7500 Fast Thermocycler (Thermo Fisher Scientific, USA). Primer concentrations, probes, and running conditions implemented in the analysis were the same as those described previously [24]. Samples were considered to be positive if they showed a Ct up to the 39th cycle (of 40 cycles) or lower [25] because it has been estimated that this protocol reproducibly detects 10 fg (4.9 genome equivalent) of genomic *C*. *burnetii* DNA up to this cycle [24]. *C*. *burnetii* DNA was used as a positive control in all the amplification protocols. Milli-Q^®^ Type I (Merck, Darmstadt, Germany) water was used as a negative control.

#### Confirmation of C. burnetii molecular detection by sequencing of 16S rRNA

Ten samples with the lowest Ct values were selected for each population from genomic DNA samples with a positive result in the molecular screening test. A nested PCR (nPCR) was performed for each of these samples, in which a partial sequence of *16S rRNA* of the *Coxiella* genus was amplified, including *C*. *burnetii* and bacteria similar to *Coxiella* (*Coxiella-like Bacteria*: CLB). The first round of PCR was performed using Cox16SF1 5′-CGTAGGAATCTACCTTRTAGWGG-3′ and Cox16SR2 5′-GCCTACCCGCTTCTGGTACAATT-3′ primers, which generated amplicons of 1321–1429 bp. Then, a second round was performed using Cox16SF1 and Cox16SR1 5′-ACTYYCCAACAGCTAGTTCTCA-3′ primers, which generated amplicons of 719–826 bp. Amplifications were performed using GoTaq® G2 Flexi DNA polymerase that contains GoTaq® G2 Flexi DNA Polymerase, 5X Green GoTaq® Flexi Buffer, 5X Colorless GoTaq® Flexi Buffer, and 25mM MgCl_2,_ and Promega dNTPs (Promega Corporation, USA) in an T100™ Thermal Cycler (Bio-Rad Laboratories, USA).The conditions used for this nPCR followed a previously described protocol [26,27]. From these samples, two samples each from cattle and livestock farmers that fulfilled the amplicon strength and quality criteria were selected for bidirectional sequencing of the molecular marker. The products selected were purified and sequenced by Macrogen (Rockville, Maryland, USA) using the same primers (Cox16SF1 and Cox16SR1) used in the second nPCR round.

#### 16S rRNA sequence analysis

DNA sequences were edited, assembled, and aligned using Geneious® 9.1.2 software (Wellesley St, Auckland, New Zealand). A BLASTn analysis was performed for each consensus sequence of *16S rRNA* obtained for each sample for comparison with the reference sequences available in the NCBI database and to identify sequences that shared the highest identity. As a complementary strategy to confirm the accuracy of the molecular diagnosis, some of the reference sequences for this marker available in GenBank were selected, and a molecular phylogenetic analysis was conducted based on the maximum-likelihood method and using PhyML plugin, available via the Geneious® software. During these analyses, a general time-reversible model was used, with gamma distribution (G) and proportion of invariable sites (I) selected by the jModelTest 2.1 tool [28]. The robustness of the phylogenetic tree was estimated using a bootstrap analysis with 1000 copies. *Legionella pneumophila* subsp. *pascullei* and *Rickettsiella melolonthae* were included as external groups. The appearance of the tree was edited using the online tool Interactive Tree Of Life (iTOL) version 4.4.2 [29].

*Factors associated with C*. *burnetii Infection*. A structured questionnaire was conducted to collect and describe some socio-demographic characteristics such as age, sex and location of the farm, hygiene-sanitary and clinical aspects about each participant. The information in the survey about cattle included age, sex, breed and medical and veterinary management history [21]. Using this information, an exploration of the possible factors associated with *C*. *burnetii* infection was performed and confirmed by molecular detection.

The results of the real-time PCR performed on *C*. *burnetii* as an initial screening strategy was defined as the outcome variable. In individuals exposed to livestock, the following associated factors were analyzed: sociodemographic characteristics, presence of domestic animals and arthropods on the property (Table 1), occupational history, types of activities performed on the farm, hygiene and sanitary characteristics, and medical history (Table 2). Conversely, the following variables were analyzed for bovine animals: age, sex, breed, place of origin, municipality of the farm, type of production, and residence time on the farm (Table 3).

**Table 1:** Descriptive, bivariate, and multivariate analyses of sociodemographic characteristics and variables related to the presence of animals and arthropods in livestock farmers’ residences associated with *C*. *burnetii* infection.

Variables	Real-time PCR for *C*. *burnetii*		Bivariate analysis			Multivariate analysis*		
	Positive N = 37	Negative N = 106						
	n (%)	n (%)	cPR	95% CI	*p* value	aPR	95% CI	*p* value
***Sociodemographic characteristics***								
*Age (years)*								
18–30	7 (18.9)	24 (22.6)	Ref.	-	0.91			
31–40	10 (27)	28 (26.4)	1.17	0.50–2.70				
41–50	10 (27)	31 (29.2)	1.08	0.46–2.52				
≥51	7 (18.9)	24 (22.6)	Ref.	-				
*Sex*								
Female	9 (24.3)	16 (15.1)	1.52	0.82–2.81	0.20	1.52	0.41–5.70	0.53
Male	28 (75.7)	90 (84.9)	Ref.	-	-	Ref.	-	-
*Municipality*								
Puerto Berrío	16 (43.2)	34 (32.1)	2.37	0.95–5.88	0.13	**2.13**	**1.10–4.11**	**0.02**
Puerto Nare	16 (43.2)	40 (37.7)	2.11	0.85–5.28		2.30	0.90–6.01	0.09
Puerto Triunfo	5 (13.5)	32 (30.2)	Ref.	-	-	Ref.	-	-
*House location*								
Rural area	31 (83.8)	88 (83)	1.04	0.49–2.22	0.91	0.59	0.23–1.53	0.28
Urban area	6 (16.2)	18 (17)	Ref.	-	-	Ref.	-	-
***Domestic animals in the house***								
Pigs	12 (32.4)	35 (33)	0.98	0.54–1.78	0.95			
Bovine animals	28 (75.7)	73 (68.9)	1.29	0.67–2.5	0.43	1.05	0.60–1.83	0.86
Hens	27 (73)	66 (62.3)	1.45	0.77–2.75	0.23	**1.47**	**1.21–1.79**	**<0.001**
Horses	25 (67.6)	59 (55.7)	1.46	0.80–2.67	0.21	**1.61**	**1.54–1.67**	**<0.001**
Sheep	1 (2.7)	6 (5.7)	0.54	0.08–3.39	0.47			
Goats	1 (2.7)	2 (1.9)	1.3	0.26–6.58	0.77			
Dogs	27 (73)	74 (69.8)	1.12	0.6–2.11	0.72			
Cats	26 (70.3)	67 (63.2)	1.27	0.69–2.35	0.44			
***Presence of arthropods***								
Cockroaches	30 (81.1)	90 (84.9)	0.82	0.41–1.64	0.59			
Flies	27 (73)	88 (83)	0.66	0.36–1.19	0.18	0.57	0.19–1.72	0.32
Fleas	18 (48.6)	53 (50)	0.96	0.55–1.67	0.88			
Acari	15 (40.5)	40 (37.7)	1.09	0.62–1.92	0.76			
Ticks	11 (29.7)	20 (18.9)	1.53	0.85–2.74	0.17	**2.36**	**1.03–5.42**	**0.04**

Ref.: reference variable defined by previous studies concerning risk factors for acquiring *C*. *burnetii* infection [10,33]; cPR: crude prevalence ratio; aPR: adjusted prevalence ratio

*Cluster-adjusted for municipality variable

**Table 2:** Descriptive, bivariate, and multivariate analyses of hygienic and sanitary characteristics and variables associated with occupational history and medical record of livestock farmers for *C*. *burnetii* infection.

Variables	Real-time PCR for *C*. *burnetii*		Bivariate analysis			Multivariate analysis**		
	Positive N = 37	Negative N = 106						
	n (%)	n (%)	cPR	95% CI	*p* value	aPR	95% CI	*p* value
***Hygienic and Sanitary characteristics***								
Hand washing before eating and cooking	35 (94.6)	93 (87.7)	2.05	0.55–7.68	0.24	1.03	0.08–13.21	0.98
Raw milk consumption	8 (21.6)	22 (20.8)	1.04	0.53–2.03	0.91	**1.47**	**1.26–1.72**	**<0.001**
Consumption of products derived from raw milk	22 (59.5)	63 (59.4)	1	0.57–1.76	0.99	0.96	0.66–1.42	0.87
Preparation of food derived from raw milk	19 (51.4)	53 (50)	1.04	0.6–1.81	0.88			
Occupational history*								
Contact with cattle	30 (81.1)	82 (77.4)	1.19	0.58–2.44	0.64			
*Time working with cattle (years)*								
≤10	9 (24.3)	37 (34.9)	Ref.	-				
11–20	12 (32.4)	32 (30.2)	1.39	0.65–2.98	0.49			
21–28	4 (10.8)	14 (13.2)	1.14	0.4–3.23				
≥29	12 (32.4)	23 (21.7)	1.75	0.83–3.69				
*Time working with cattle (hours/day)*								
≤1	11 (29.7)	26 (24.5)	Ref.					
2–8	23 (62.2)	72 (67.9)	0.81	0.44–1.5	0.81			
≥9	3 (8.1)	8 (7.5)	0.92	0.31–2.71				
Direct contact with cattle fluids	22 (59.5)	73 (69.5)	0.73	0.42–1.27	0.26	0.95	0.62–1.45	0.82
Presence of ticks in the farm	32 (86.5)	90 (84.5)	1.1	0.49–2.5	0.82			
History of tick bites	21 (56.8)	70 (66)	0.75	0.43–1.31	0.31			
History of presence of immature forms of ticks	18 (48.6)	58 (54.7)	0.84	0.48–1.45	0.52			
***Type of activity performed***								
Livestock milking	13 (35.1)	50 (47.6)	0.68	0.38–1.22	0.19	0.95	0.57–1.56	0.83
Livestock enclosure	15 (40.5)	49 (46.7)	0.83	0.47–1.47	0.65			
Livestock bathing	8 (21.6)	27 (25.7)	0.84	0.43–1.67	0.62	1.09	0.39–3.08	0.87
Vaccination	15 (40.5)	46 (43.8)	0.91	0.51–1.59	0.73			
Fumigation	11 (29.7)	30 (28.6)	1.04	0.57–1.91	0.89			
Livestock slaughtering	2 (5.4)	15 (14.3)	0.42	0.11–1.59	0.15	0.42	0.04–4.58	0.48
Offspring care	2 (5.4)	3 (2.9)	1.57	0.52–4.76	0.47			
Farm enclosure	5 (13.5)	31 (29.5)	0.46	0.19–1.09	0.06	0.43	0.11–1.68	0.23
Clinical record*								
Blood transfusion	2 (5.4)	10 (9.4)	0.62	0.17–2.28	0.45			
Fever	12 (32.4)	37 (34.9)	0.92	0.51–1.67	0.78			
Chills	11 (29.7)	36 (34)	0.87	0.5–1.54	0.64			
Fatigue	4 (10.8)	17 (16.2)	0.7	0.28–1.77	0.43			

Ref.: reference variable defined by previous studies concerning risk factors for acquiring *C*. *burnetii* infection [10,33]; cPR: crude prevalence ratio; aPR: adjusted prevalence ratio

*Corresponds to the self-report performed by each study participant

**Cluster-adjusted by municipality variable

**Table 3:** Descriptive, bivariate, and multivariate analysis of sociodemographic characteristics and factors associated with the presence of *Coxiella* bacteria in cattle.

Variables	Real-time PCR for *C*. *burnetii*		Bivariate analysis			Multivariate analysis**		
	Positive N = 38	Negative N = 154						
	n (%)	n (%)	PR	95% CI	*p* value	PR	95% CI	*p* value
***Age (months)***	*** ***	*** ***						
≤9	7 (18.4)	44 (28.6)	Ref.	-	-	Ref.	-	-
10–20	9 (23.7)	37 (24)	1.43	0.58–3.52	0.6	1.03	0.30–3.52	0.97
21–60	12 (31.6)	41 (26.6)	1.65	0.71–3.86		1.07	0.08–15.13	0.96
≥61	10 (26.3)	32 (20.8)	1.74	0.72–4.16		0.84	0.10–6.85	0.87
***Sex***	*** ***	*** ***						
Female	22 (57.9)	74 (48.1)	1.38	0.77–2.45	0.28	1.01	0.42–2.42	0.99
Male	16 (42.1)	80 (51.9)	Ref.	-	-	Ref.	-	-
Breed*	*** ***	*** ***						
Mixed breed	9 (23.7)	41 (26.6)	0.9	0.23–3.55	0.87			
*Bos indicus*	17 (44.7)	74 (48.1)	0.93	0.25–3.47				
*Bos taurus*	10 (26.3)	31 (20.1)	1.22	0.32–4.71				
Colombian Creole breeds	2 (5.3)	8 (5.2)	Ref.	-				
***Place of origin***	*** ***	*** ***						
Born on the farm	30 (78.9)	118 (76.6)	1.11	0.55–2.25	0.76	0.95	0.50–1.81	0.87
Born on another farm	8 (21.1)	36 (23.4)	Ref.	-	-	Ref.	-	-
***Municipality where farm is located***								
Puerto Berrío	15 (39.5)	49 (31.8)	**0.35**	**0.15**–**0.84**	**0.03**	0.95	0.74–1.24	0.72
Puerto Nare	6 (15.8)	58 (37.7)	0.88	0.48–1.61		**0.39**	**0.37–0.41**	**<0.001**
Puerto Triunfo	17 (44.7)	47 (30.5)	Ref.	-		Ref.	-	-
***Type of production***	*** ***	*** ***						
Milk	15 (39.5)	44 (28.6)	3.05	0.44–20.95	0.46			
Meat	17 (44.7)	81 (52.6)	2.08	0.30–14.28				
Offspring	5 (13.2)	18 (11.7)	2.61	0.34–19.87				
Double purpose	1 (2.6)	11 (7.1)	Ref.	-				
***Time of residence of cattle on the farm(months)***								
≤8	7 (18.4)	46 (29.9)	Ref.	-	-	Ref.	-	-
9–14	9 (23.7)	34 (22.1)	1.59	0.64–3.91	0.17	1.23	0.75–1.99	0.41
15–48	9 (23.7)	45 (29.2)	1.26	0.51–3.14		1.14	0.17–7.49	0.89
≥49	13 (34.2)	29 (18.8)	2.34	1.03–5.35		**2.28**	**1.03–5.20**	**0.04**

Ref.: reference variable as defined by statistical criteria (the one with the lowest report occurrence).

*For better presentability, breeds were categorized as follows: *Bos indicus*: cattle of Indian origin, also known as zebu cattle, breeds such as Brahman, Cebú, Gyr and Simbrah were included; *Bos taurus*: cattle of European origin, breeds such as Holstein, Jersey, Ayrshire, Simmental, Brown Swiss, Normande, Senepol, and Swedish Red were included; Colombian creoles: BON and Romosinuano.

**Cluster-adjusted by municipality variable

Relative and absolute frequencies were calculated for qualitative variables using SPSS^®^ statistical software (SPSS^®^ Inc., Chicago, USA). When analyzing factors associated with *C*. *burnetii* infection, the crude prevalence ratio (cPR) and adjusted prevalence ratio (aPR) and its corresponding confidence interval (CI) at 95% were estimated using Poisson regression with forward selection method. Moreover, standard errors were adjusted by cluster effect for each municipality since farms, individuals, and cattle present a municipality-determined natural grouping. Analyses were performed using Stata^®^ software (Lakeway Drive, Texas, USA).

In the multivariate models used for both populations, the variables were included because they met the statistical criterion of a *p* value of <0.25 after performing bivariate analysis, or because they play an important role in disease transmission or are risk factors that were previously described [5,8,30–33]

## Results

### Demographic characteristics of the study populations

Most livestock farmers in the study were men (82.5%) and aged 41–50 years (28.7%). Regarding place of residence, 83.2% of participants lived in rural areas, and the main activities performed on the farms were livestock enclosure activities (44.8%) and milking activities (44.1%). Moreover, high presence of ticks, history of tick bites, and presence of tick immature stages were reported by 85.3%, 62.9%, and 53.1% of respondents, respectively. Domestic animal ownership was also reported by respondents, with dogs (70.6%) and livestock (70.6%) being the most owned. Preparation of food derived from raw milk was reported by 50.3% of individuals, and regular consumption of raw milk was reported by 59.4% of respondents.

Conversely, 50% of the cattle analyzed were male and 21–60 months old. In terms of breeds, 43.2% were *Bos indicus*, 34.4% were mixed breed, 18.2% were *B*. *taurus*, and 4.2% were Colombian Creole breeds.

### Molecular detection and analysis of 16s rRNA sequences

*C*. *burnetii* was detected among 25.9% (37/143) of livestock farmers. Among individuals who tested positive for the bacterium detected using real-time PCR, 86.4% (32/37) resided in Puerto Berrío and Puerto Nare (Tables 1 and 2).

The rate of *Coxiella* frequency as determined by molecular detection in cattle was 19.5% (38/192). The highest one was found in Puerto Triunfo, where 44.7% (17/64) of samples were positive; the majority of positive results occurred in females (57.9%), which were born on the farm where the present study was performed (78.9%), were intended for meat production (44.7%), and spent over 49 months of residence on the farm (34.2%) (Table 3).

Four sequences of the partial fraction of *16S rRNA* were collected for this analysis, two from livestock farmer samples (H301 and H316 codes) and two from cattle samples (B237 and B285 codes). These sequences were deposited in GenBank under accession numbers MN540436, MN540437, MN540442, and MN540443.

**Fig 2:**
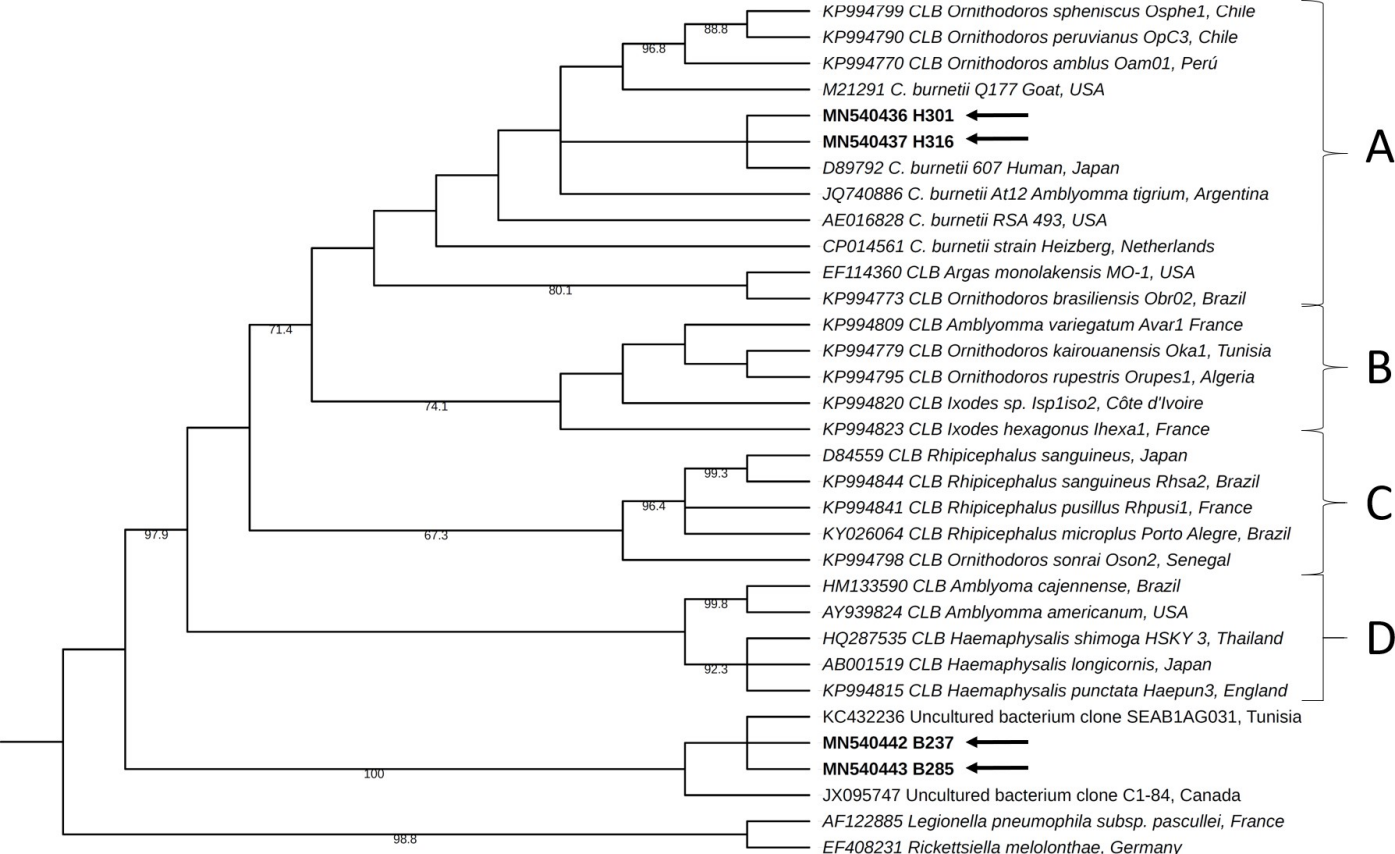
Phylogenetic tree constructed using maximum-likelihood based on partial sequences of *16S rRNA* for *Coxiella* genus.

BLASTn revealed that the sequences belonging to samples collected from two livestock farmers showed high identity percentages (99%–100%) with *C*. *burnetii* and were grouped within A clade in the phylogeny analysis, a group where this species had been previously described within the *Coxiella* genus [27]. In contrast, the sequences collected from two of the positive cattle samples showed the highest identity percentages, ranging from 98% to 100%, with DNA sequences reported in GenBank as nonculturable bacteria from environmental studies, as well as identity percentages of 89%–90% with different reference sequences of *Coxiella* sp. Consistent with the molecular phylogeny analysis results, the sequences obtained from cattle were grouped in a separate clade from groups A to D previously reported for *Coxiella* genus (Fig 2).

All four clades previously reported for the *Coxiella* genus [27] are labeled with letters A to D. The *C*. *burnetii* group is included within clade A. The labels for each reference sequence include the GenBank accession number and specify the country of origin. Its branches have numbers indicating bootstrap support (out of 1,000 repetitions), with values ranging from 60% to 100%. The sequences obtained for the present study are indicated by arrows. CLB: *Coxiella*-like bacteria.

### Factors associated with the detection *Coxiella*

In the multivariate model, the occurrence of *C*. *burnetii*, as determined by molecular detection, among livestock farmers was found to be higher in Puerto Berrío compared with Puerto Nare and Puerto Triunfo, and was related to the presence of hens, horses and ticks in the place of residence. Moreover, the consumption of raw milk was also identified as a related factor (Tables 1 and 2).

Among cattle, the frequency of bacteria of the genus *Coxiella*, as determined using real-time PCR, was lower in Puerto Nare than in Puerto Triunfo and Puerto Berrío and higher in cattle with a longer residence time on the farm (i.e., ≥49 months) (Table 3).

## Discussion

The molecular detection results revealed the presence of *C*. *burnetii* in livestock farmers (25.9%) and a bacterium associated with the *Coxiella* genus in cattle (19.5%) in the Magdalena Medio region of Antioquia, Colombia. Moreover, the factors associated with molecular detection of this species were identified in both humans and cattle.

For livestock farmers and cattle from the Magdalena Medio region of Antioquia, the occurrence of *C*. *burnetii* as determined by molecular detection in the present study is high compared with the results from previous molecular studies. For instance, in a study conducted in Iran and published in 2018, 9.8% of 173 (17/173) seropositive individuals from populations with a high risk of infection (slaughterhouse employees, butchers, farmers, and veterinaries) were found to have DNA from this bacterium [34]. In a study performed on livestock from farms in South Korea in 2017, *C*. *burnetii* was identified by PCR only in 1.5% (11/736) of cattle and in 10.5% (77/736) by serology [31].

The analysis of partial *16S rRNA* sequences from livestock farmers and cattle samples was essential, and they were initially classified as positive for *C*. *burnetii* infection using real-time PCR. In the samples collected from two of the livestock farmers, *C*. *burnetii* infection was confirmed through an analysis of the partial sequences of the *16S rRNA* marker, whereas in the samples collected from two of the cattle, a bacterium related to the *Coxiella* genus was detected whose taxonomic classification is yet to be clarified. Thus, the identification of *C*. *burnetii* infection among livestock farmers emphasizes the potential importance of this bacterium as a human pathogen that causes short- and long-term morbidity in the study area [6]. Regarding the results obtained by the analysis of sequences collected from cattle samples, various bacteria defined as *Coxiella-*like bacteria have been identified over the past decades: CLB both in soft and hard ticks, which have been grouped within the *Coxiella* genus, but are genetically different from *C*. *burnetii*. A study performed in South Korea identified *16S rRNA* sequences in five horses that corresponded to CLB bacteria [35]; these were classified into a new clade of the *Coxiella* genus, as was the case in the present study. This suggests that the diversity within the *Coxiella* genus can be wider than presently known and also that it is essential to conduct studies to provide a taxonomic definition of these microorganisms and enhance epidemiological surveillance because their pathogenic potential is unknown even though studies based on molecular phylogeny have suggested that *C*. *burnetii* shares a common ancestor with these CLB, which are considered to be endosymbionts of ticks [27]. More importantly, we have found *Coxiella* species from cattle and humans that fell into two different clades suggesting that there might be an unidentified reservoir.

The results of the present study, together with those previously obtained by Eraso *et al*. [21], concerning this same area and study groups, suggest that conducting a more active search for this bacterium as an etiological agent of diseases, in both humans and cattle, and focused in people with high occupational risk is important. In addition, epidemiological surveillance of this zoonotic agent is important in both the livestock scenario, as well as in hospitals.

Individuals who reported living with hens and horses had a higher frequency of *C*. *burnetii*. In a previous study performed in South Korea, 816 blood samples from horses were analyzed, this bacterium was identified by immunoassay in 11 (1.3%) of the population; additionally, researchers found a bacterium of the genus *Coxiella* in six horses using molecular techniques to identify the 16S rRNA gene [35]. Moreover, while searching for additional *C*. *burnetii* hosts, Roest *et al*. evaluated placentas obtained either during parturition or abortions of cats, dogs, sheep, goats, pigs, bovines, and horses and identified this bacterium in 8% (3/39) of equine placentas [36], suggesting that conducting studies to assess whether horses can be relevant indicators, hosts, reservoirs, or sentinel animals during the transmission cycle of this bacterium is important [30].

Regarding the presence of ticks in the place of residence, it is known that ticks are vectors of many pathogenic microorganisms [37]. Its role as a source of infection for this bacterium is still debated; however, several studies have detected it in different tick genera. In 2019, it was identified as the most frequent pathogen in 130 tick pools from South Africa, via PCR sequencing with a frequency of 9.2% [38]. Another study also used PCR sequencing to identify the DNA of this bacterium in ticks collected in Iran, which included three pools of *Hyalomma anatolicum* (each with five female ticks) and one of *Rhipicephalus sanguineus* (six ticks). These results suggest that ticks could be a possible vector in the natural history of this microorganism [39]. Moreover, previous studies assessed experimental transmission of *C*. *burnetii* from infected to uninfected guinea pigs via tick bite, and this transmission method has been demonstrated by *Ixodes holocyclus*, *Haemaphysalis bispinosa*, and *Rhipicephalus sanguineus* [40]. However, whether the form of infection in a livestock context is direct (i.e., from a tick bite) or indirect (i.e., by inhaling its fecal particles) is still unknown. To address this, a study published in 2020 sought to analyze the uptake, survival and transstadial transmission of *C*. *burnetii* in ticks, as well as excretion of the bacteria via feces under controlled laboratory conditions. The study demonstrated that adult ticks that became infected with *C*. *burnetii* as nymphs contain viable bacteria and excrete them via feces while feeding on non-infected blood. This excretion of bacteria followed a different time course and contained higher concentrations than non-infected ticks feeding on infected blood [41]. The information on the detection of this microorganism in these types of ectoparasites and vectors as well as in the context of livestock is still limited, which is why studies are needed to evaluate the animals, humans, and ticks present in the same context, to elucidate the possible role of each of these actors in the transmission of this zoonotic agent [42].

Moreover, as previously mentioned, a relationship between the occurrence of *C*. *burnetii* and the presence of hens in the individuals’ place of residence was observed, which is consistent with the findings from a serological study by Eraso *et al*. [21] in this same population group. Although the importance of *C*. *burnetii* as an infectious agent in hens had not been previously evaluated, a study in Japan assessed the presence of this microorganism in samples collected from chicken eggs and commercial mayonnaise and reported an occurrence of 4.2% and 17.6%, respectively. Moreover, the real-time PCR used in that study allowed for the quantification of *C*. *burnetii* cells, and the estimated values ranged from 10^4^ to 10^6^ per egg [43]. Because it is known that the infectious dose (ID50) in humans is 1.18 bacteria [44], such a high inoculum value in these types of poultry products suggests that these can serve as potential infection source for the overall population, and this aspect should be evaluated.

When considering the general life cycle of *C*. *burnetii*, it is observed that ruminants are one of the main reservoirs of this microorganism; however, because of the findings associated with poultry, further research on the presence of *C*. *burnetii* in the poultry chain should be conducted. Research should also focus on other non-domestic animals, such as rodents, that are usually present in livestock production systems and could play an important role as reservoirs of this bacteria and as a source of infection for other animals and humans, either through biologic fluids or through consumption of infected food [6].

We identified the consumption of raw milk as a factor associated with the increase in the frequency of molecular detection of the bacteria. In a previous serological study conducted in this same population of farmers in Magdalena Medio [21], an association was observed between the consumption of raw milk derivatives (OR: 4.04; 95% CI: 1.58–10.34) and seropositivity to *C*. *burnetii*. Among the food products of animal origin, raw milk is considered the most important source of infection by this bacterium because it is excreted in the milk of infected animals (e.g., cattle, sheep and goats), whether they show clinical signs of infection or not [45]. In a cross-sectional study conducted in Iran, 48 samples of raw milk were randomly collected at a retail market; after nested PCR was conducted to detect the DNA of this microorganism, 13 samples (27.1%) were found to be positive [46]. Moreover, there is epidemiological evidence of Q fever cases where the consumption of unpasteurized milk was the most likely source of infection. One of the reports was made in Michigan, USA, in 2011, and involved five people with positive serological tests for *C*. *burnetii* that suggested a diagnosis of Q fever, where the only source of infection that was verified in common was the consumption of raw milk [47]. Although there is evidence of the possible transmission of this bacterium via this type of food, these aspects of the natural history of the transmission of this microorganism have not yet been clarified, which highlights that conducting new studies to assess possible ways of acquiring the infection in our environment is important. By performing a more complete epidemiological surveillance and including molecular analyses, these studies will provide greater sensitivity than previously conducted studies.

Regarding the cattle analyzed in the present study, we observed that the main factor associated with the detection of bacteria of the *Coxiella* genus was when the time of residence on the farm was greater than or equal to 49 months. According to a study published in 2011, where the dynamics of infection within dairy farms were analyzed through serological tests over time, a relationship between seropositivity and the age of the animal was observed, which suggests that *C*. *burnetii* may cause persistent infections in cattle [48]. Furthermore, Carbonero *et al*. identified age of the cattle (OR: 1.01; CI95%: 1,006–1.014), feeding of calves with milk replacers (OR: 1.94; 95% CI: 1.1–3.3), bovine respiratory syncytial virus seropositivity (OR: 1.54; 95% CI: 1.1–2.3), and disinfection of the umbilical cord (OR: 0.60; 95% CI: 0.4–0.9) [49] as associated factors in dairy and mixed cattle farms from Ecuador, so the results of the present study are supported by those of previous studies [50,51]. Furthermore, an explanation can be found in the natural history of this infection, since infection with this bacterium is known to be highly related to the time and duration of exposure to animal reservoirs [52], and as known in the livestock context, cattle can interact with various wild animals, besides other domestic animals [53]. This bacterium was also found in wild rodents in Brazil, with a frequency of 4.6% (6/131) [54], demonstrating the need to consider new, more holistic studies, that evaluate other possible reservoirs and ways of acquiring the infection in our environment, especially in agricultural contexts [55].

A limitation of the present study was that the sociodemographic and epidemiologic surveys used to identify factors associated with *C*. *burnetii* presence were reliant on participants’ self-reporting; therefore, a memory bias may have been introduced. Moreover, cross-sectional studies such as this do not allow for the evaluation of the causality of the analyzed variables since the temporality criterion is not fulfilled; nevertheless, this type of research helps to establish hypotheses for future cohort studies. Furthermore, because the sequencing of positive cattle samples for the initial molecular detection was performed on a limited subset, the *C*. *burnetii* infection rate could not be accurately estimated in the present study; however, *Coxiella* genus bacteria are important to evaluate considering their potential role as zoonotic agents.

In conclusion, the presence of *C*. *burnetii* in livestock farmers (25.9%) and *Coxiella* genus bacteria in cattle (19.5%) from the Magdalena Medio region of Antioquia suggests circulation of this bacterium among livestock in this area. The mismatch of clades between *Coxiella* identified in livestock and humans suggests that other farm associated animals may be involved in the transmission. Therefore, it is important to consider the potential role that *C*. *burnetii* may play as an etiological agent of acute febrile syndrome, pneumonia, and hepatitis, as well as endocarditis, vascular infection, and post-infectious fatigue syndrome in patients from this area. A differential diagnostic in this type of population should also be implemented. Moreover, the exploratory analysis of factors associated with *C*. *burnetii* detection in livestock farmers performed in the present study highlights that performing studies that are aligned with the global One Health strategy, in which greater interdisciplinarity is suggested to integrate human and animal healthcare with the ecosystems in which they coexist, is important [56]. Based on this approach, any zoonotic agent should be studied thoroughly while considering all factors that perpetuate its transmission cycle.
